# Variability of cassava (*Manihot esculenta* [Cranz]) genotypes for nutritional composition in Southwestern Ethiopia

**DOI:** 10.1038/s41598-026-54163-2

**Published:** 2026-05-29

**Authors:** Neim Semman Abadura, Ismail Yusuf Rabbi, Tewodros Mulualem Beyene, Wosene Gebresellassie Abtew, Abush Tesfaye Abebe

**Affiliations:** 1 Ethiopian Institute of Agricultural Research, Addis Ababa, Ethiopia; 2 Jimma University Colleges of Agriculture and Veterinary Medicine, Jimma, Ethiopia; 3 International Institute of Tropical Agriculture, Ibadan, Nigeria

**Keywords:** Cassava, Biochemical, Biofortified, Coefficient of variance component, Multivariate, Genetics, Plant sciences

## Abstract

The study utilized a total of 100 cassava genotypes sourced from IITA, Nigeria (biofortified genotypes) and locally collected landraces of Ethiopia, conserved at Jimma Agricultural Research Center. So, understanding variability in nutritional composition can guide future genetic improvement and support the development of nutritionally enhanced cassava varieties. The objective of the study was to assess variability of cassava genotypes-based their nutritional compositions analysis. The analysis of variance (ANOVA) revealed highly significant differences among the studied genotypes for all traits. The mean separation analysis revealed largest values of Beta carotene was recorded with genotypes G79 (18.91) and G106 (17.82%) whereas maximum starch content of 77.79 and 77.22% were recorded with genotype G33 and G29respectively. The maximum dry mater content was recorded with G100 (75.93%) and G128 (71.63%). Correlation analyses revealed highly significant and positive associations between organic matter and dry matter contents (0.98***). Ash content positively correlated with beta-carotene (0.28**) but negatively correlated with OM (-0.33***). However, starch positively correlated with OM (0.135) and DMC (0.124) and poor but negative correlated with protein. These association indicated that traits influence one another which might be due to physiological trade-off or the increment of one trait cause to the increment of another trait. PCA further revealed substantial variation among genotypes, where the first three PC with Eigen values > 1 explained 68.00% of the total variation. Dry matter, moisture content and organic matter were major contributors to Dim-1, while protein and nitrogen contributed to Dim-2, whereas tannin showed a slight negative association with dimension 2. Cluster analysis grouped the studied genotypes into six distinct clusters, each characterized by specific trait combinations. Generally, the results of the study provided concrete information for future cassava breeding program in Ethiopia.

## Introduction

 Cassava (*Manhot esculenta* [Cranz]) is an evergreen shrubby root crop grown in the tropical and sub-tropical regions of the world. The crop is characterized by its high yield and bankable underground root harvested for consumption as food^1^. It ensures food security for millions of people, mainly in developing countries of Sub-Saharan countries^2^. It is a great source of starch for human food and animal feed and provides high nutritional values to the urban and rural population^3–5^. Also, its production is intended for human consumption, sources of income and industrial production of starch, flour, beverages, animal feeds, biofuels and textiles, contributing to its global importance^6^.

It is extensively produced in SSA ensuring food security for the low-income community, and hence considered “poor people crop” and also called “dangerous crop” due to the low quality and quantity of protein and the presence of cyanogenic glycoside^7,8^. The regions where cassava is grown are characterized by widespread deficiency of minerals and vitamins^5^. This resulted in wider suffering and death of people due to hidden hunger, which needs to be addressed through assessing the nutritional composition of the available germplasm and identifying nutritional dense varieties through breeding, as well as supplementing with nutritionally enhanced diets. In most of the root and tuber crops producing areas, farmers habitually supplement their food with protein-rich foods, such as pulse crops, like faba beans, peas, and also animal-based foods^9^.

Cassava tubers are rich in starch (35%) but poor in protein content, around 1.1%, while the ash content ranged from 4.23 to 8.32%^3^. The nutritional composition of cassava varies depending on varieties, plant age, plant parts utilized, environment and soil types^10,11^. assessed six cassava varieties for their proximate composition and reported moisture, protein and ash contents in the ranges of 10.43–11.18, 1.21–1.87, 1.21–1.78, and 0.03–0.60% respectively. Additionally^8^, analyzed the biochemical, mineral and proximate content of 19 cassava varieties and reported starch content ranging from 24 to 40.91% and nitrogen content ranging from 0.41 to 0.97% in the tubers. Another crucial nutritional composition of cassava tubers is β-carotene content, which has an essential nutritional contribution in addressing health problems caused by vitamin A deficiency, which affect the majority of smallholder farmers in developing countries. β-carotene content is essential for human health, as β-carotene is the precursor of provitamin A^12^. Despite cassava roots are rich sources of carbohydrate, negligible amount of protein and β-carotene contents reported in white cassava tubers^13^. As a result, genetic modification of cassava roots was applied conventionally as well as in modern techniques to produce high beta-carotene content cassava tubers.

In Ethiopia, cassava is widely cultivated in the South and Southwest parts of Ethiopia in areas where smallholder farmers are densely populated. The cassava varieties in the hands of farmers are local landraces with low yield potential and low nutritional content with white storage root pulp. Berhanu et al. (2022) studied the biochemical compositions of 64 cassava genotypes in Ethiopia and reported moisture contents ranging from 4.8% to 10.1%, ash (2.10% to 3.96%), carbohydrate (81.3 to 87.8%) and protein (1.3 to 2.5%) contents. The crop provides high nutritional value, where the root is the chief sources of carbohydrate^14^. Recently, a hybrid of biofortified cassava genotypes was introduced from IITA cassava breeding program to broaden the cassava germplasm bases of the cassava improvement program of the Jimma Agricultural Research Center (JARC) to be utilized for the genetic improvement of cassava. Additionally, there were cassava genotypes collected from the different cassava growing regions of Ethiopia and cassava germplasm introduced from IITA long years ago and conserved at Jimma Agricultural Research Center. However, some of the major nutritional composition of their storage root has not so far been assessed. Hence, this study was aimed at assessing the biochemical composition of these materials.

## Materials and methods

### Experimental materials

This study made use of 100 cassava genotypes (Table 1) selected based on the characterization and cluster analyses results of the 182 cassava genotypes previously studied for agro-morphological traits^15^. Representative genotypes were selected from each cluster based on root pulp color, yield and other traits (Fig. 4).

### Sample preparation

During harvesting, root samples from the selected genotypes were collected into small polyethene containers. As described in^15^, the study was conducted at Jimma Agricultural Research Center (JARC) and Mettu but the samples were collected from the trial conducted at Jimma. The sampled roots were properly washed using clean water to remove soil and other contaminating debris. The roots were devoid of their cortex/peeled and the clean fleshy root pulps were immediately chopped, while recording the color of each pulp. From the chopped root pulp of each genotype, 100 gm sample (Fig. 4) was oven dried at 60 °C for 72 h as suggested by^16^, until constant weight was achieved. Then, the oven-dried flour samples were weighed using a sensitive balance to determine the dry matter and moisture content. The oven-dried flour samples were sent to the IITA Food and nutrition lab. for the analysis of ash, tannin and starch content. Similarly, samples were sent to the Ethiopian Institute of Agricultural Research Food and Nutrition Analysis Laboratory to analyze protein and beta-carotene contents. All the analyses were performed in duplicates.

### Compositional determination methods

#### Dry matter and moisture content determination

The dry matter was determined using an initial 100 g weight (Wi), and the samples were re-weighted (W_f_) after oven drying. Then, the percentage dry matter (%DM) and moisture contents (%MC) were calculated using the following formula.

$$\:\mathrm{\%}\mathrm{D}\mathrm{M}=\frac{{W}_{f}}{{W}_{i}}\mathrm{x}\:100$$

The percentage moisture content was calculated as:

$$\:\mathrm{\%}\mathrm{M}\mathrm{C}=\frac{{W}_{wt-{D}_{wt}}}{{W}_{wt}}\:\mathrm{x}\:100$$

### Ash content determination

Two grams of cassava flour was weighed into a porcelain crucible (Wc) and the samples were transferred into a muffle furnace and burnt at a temperature of 550 0 C for four hours. After the samples were burned, the weight of the crucible containing ash was recorded (Wb). Then, the percentage ash was calculated using the formula used by^16^:

$$\:\mathrm{\%}\mathrm{A}\mathrm{s}\mathrm{h}\:=\:(\mathrm{W}\_\mathrm{b}-\mathrm{W}\_\mathrm{c})/\mathrm{W}\_\mathrm{s}\:\mathrm{x}\:100$$

Where Wb = crucible + ash, in g.

Wc = weight of empty crucible, in g, and W_s is the weight of the sample.

### Organic matter (OM)

The OM contents of the samples were determined by subtracting the percentage ash content from the %DM as:

$$\:\mathrm{\%}\:\mathrm{O}\mathrm{M}\:=\:\mathrm{\%}\:\mathrm{D}\mathrm{M}\:-\:\mathrm{\%}\:\mathrm{a}\mathrm{s}\mathrm{h}$$

### Crude protein and nitrogen content determination

The determination of crude protein and beta-carotene content was done using HPLC machine.

#### Protein content determination

Total protein was extracted following the (1976)^17^ assay with slight modifications. Approximately 1 g of the powdered sample was mixed with 10 mL of extraction buffer (50 mM phosphate buffer, pH 7.0, containing 0.1% Triton X-100). The mixture was homogenized and centrifuged at 10,000 rpm for 15 min at 4 °C. The supernatant was collected and filtered through a 0.45 μm membrane filter before injection into the HPLC system. Protein separation and quantification were carried out using a reversed-phase high-performance liquid chromatography (RP-HPLC) system equipped with a C18 column (250 mm × 4.6 mm, 5 μm particle size, mobile phase in which solvent A (0.1% trifluoroacetic acid (TFA) in water) and Solvent B (80% acetonitrile with 0.1% TFA), Gradient elution of 5% to 60% B over 30 min, Flow rate (1 mL/min), Column temperature of 30 °C with Detection wavelength of 220 nm used.

#### Protein quantification

The protein content was quantified using a BSA (Bovine Serum Albumin) standard curve prepared in the range of 10–100 µg/mL. Peak areas corresponding to protein fractions were integrated, and protein concentrations were determined by comparing them with the BSA standard curve.

#### Beta-carotene determination

Beta carotene content of cassava flour of the studied materials was analyzed using the High-Performing Liquid Chromatography (HPLC) method, following the steps listed in^18^, where 2 g of flour samples were uniformly mixed into 250 ml a round-bottom flask. Then water was added to the sample and dispersed. Then, 100 mg of hydroquinone, 40 ml ethanol, and 10 ml KOH solution were added, and the flask was placed in a hot water bath and continuously shaken for 30 min. The solvent was cooled, and the stirring and saponification process were carried out for 30 min. After saponification, 50 ml of Petroleum ether was added and gently shaken. The second extraction was done, and the extract was washed with water. Then, the extract was filtered and dehydrated with sodium sulfate. The filter collected, evaporated using a rotary evaporator at < 40 °C under nitrogen, and quantified for beta carotene content.

#### Starch extraction

Starch extraction and determination were conducted using the HPLC procedures followed by^19^. In this procedure, glucose was hydrolyzed and starch content was derived from the hydrolyzed glucose content of food using the following conversion factor:

$$\:\%\mathrm{S}\mathrm{t}\mathrm{a}\mathrm{r}\mathrm{c}\mathrm{h}\:=\:\mathrm{\%}\mathrm{G}\mathrm{l}\mathrm{u}\mathrm{c}\mathrm{o}\mathrm{s}\mathrm{e}\times\:0.9$$

#### Tannin extraction

Tannin content of cassava flour was analyzed using High-Performing Liquid Chromatography (HPLC) based method. For the analysis, 1 g of dried sample was weighed using a sensitive balance and extracted using 80% methanol by sonication for 30 min. The solvent was centrifuged and filtered using 0.45 μm membrane. Gradient elution was carried out at 90% A / 10% B, increasing B to 50% over 30 min. Detection of tannin at UV of 280 nm following the procedure of^20^.

**Table 1:** Lists of cassava genotypes analyzed for biochemical contents.

T/*N*	Code	Genotypes	T/*N*	Code	Genotype	T/*N*	Code	Genotype	T/*N*	Code	Genotypes
1	G2	IITA1/2	26	G44	IITA5/3	51	G86	IITA9/6	76	G124	1,011,224
2	G3	IITA1/3	27	G47	IITA5/6	52	G87	IITA9/7	77	G125	5048-33
3	G5	IITA1/5	28	G48	IITA5/7	53	G89	IITA9/9	78	G127	1,050,125
4	G6	IITA1/6	29	G49	IITA5/8	54	G92	IITA10/3	79	G128	1,980,510
5	G8	IITA1/8	30	G50	IITA5/10	55	G93	IITA10/4	80	G129	1,062,630
6	G9	IITA1/9	31	G51	IITA5/11	56	G94	IITA10/5	81	G131	10,540
7	G10	IITA1/10	32	G53	IITA6/2	57	G97	IITA10/8	82	G134	1,070,337
8	G12	IITA2/2	33	G55	IITA6/4	58	G98	IITA10/9	83	G135	1,070,593
9	G13	IITA2/3	34	G57	IITA6/6	59	G99	IITA10/11	84	G137	Qulle
10	G14	IITA2/4	35	G58	IITA6/7	60	G100	IITA10/12	85	G139	MM 96/9308
11	G16	IITA2/6	36	G60	IITA6/9	61	G103	IITA10/15	86	G140	Korre- dhaske-8
12	G19	IITA2/9	37	G62	IITA6/11	62	G104	IITA11/1	87	G142	5338-19
13	G20	IITA2/10	38	G64	IITA6/13	63	G105	IITA11/2	88	G143	AWC-4
14	G21	IITA2/11	39	G65	IITA6/14	64	G106	HawK12/1	89	G144	WALAMO
15	G23	IITA2/18	40	G66	IITA7/1	65	G107	HawK12/2	90	G149	1,010,085
16	G24	IITA2/14	41	G68	O-2	66	G108	HawK12/3	91	G151	Hawassa − 04
17	G25	IITA2/15	42	G69	O-3	67	G109	HawK12/4	92	G152	AAGT-108
18	G26	IITA2/16	43	G72	O-6	68	G111	HawK12/6	93	G154	196/624
19	G28	IITA3/1	44	G73	O-7	69	G113	HawQ13/2	94	G157	7,070,824
20	G29	IITA3/2	45	G75	IITA8/2	70	G116	HAWQ13/5	95	G158	Wajo Bohe
21	G33	IITA3/6	46	G76	IITA8/3	71	G117	HAWQ13/7	96	G159	AAGT-191
22	G34	IITA3/7	47	G77	IITA8/4	72	G118	HAWQ13/8	97	G162	1554
23	G35	IITA4/1	48	G79	IITA8/6	73	G121	156	98	G163	1038
24	G38	Wh-29	49	G80	IITA8/7	74	G122	AWC-5	99	G169	5648-50
25	G40	Wh-12	50	G82	IITA9/2	75	G123	MM96/3280	100	G179	M94/0117

### Data analysis

#### Analyses of variances

The analysis of variances was performed using the ‘aov()’ function of the ‘agricolae’ package of R software version 4.4.2^21^. The following model of CRD was used:

y_ij_= X_t_+ y_r_ + E, i and j represent the effect of treatment and replication respectively.

Significant differences among samples were determined using one-way ANOVA (*p* < 0.001).

### Multivariate data analyses

Based on test of significance differences, the multivariate analyses such as correlation, principal component and cluster analyses were performed using R software version 4.4.2.

#### Correlation analysis

The associations among the different traits were analyzed using facto-extra package of R-software, with Pearson correlation model^22^.

$$r=\frac{{\sum\nolimits_{{i=1}}^{n} {{\mathrm{(}}{x_i} - \bar {x}){\mathrm{(}}{y_i} - \bar {y})} }}{{\sqrt {\sum\nolimits_{{i=1}}^{n} {{{{\mathrm{(}}{x_i} - \bar {x})}^2}} } \;\sqrt {\sum\nolimits_{{i=1}}^{n} {{{{\mathrm{(}}{y_i} - \bar {y})}^2}} } }}$$

where.

• r = Pearson correlation coefficient.

• xi, yix_i, y_ixi, yi: individual observations of variables X and Y.

• xˉ,yˉ\bar{x}, \bar{y}xˉ,yˉ: means of X and Y.

• nnn: number of paired observations.

##### Cluster analysis

The scaled data was subjected to R-software of hcut() function to group genotypes based on their similarity, where “cluster” packages were used and the dendrogram was displayed using fviz_dend() function using “ggplot” package (Kassambara and Mundt, 2020)^22^.

##### Principal component analysis (PCA)

The PCA is a method used to signify the traits that contributed more to the total variations in the germplasm studied. It is a technique for reducing the dimensionality of such datasets, increasing interpretability, but at the same time minimizing information loss by creating new uncorrelated variables that successively maximize variance. In a similar approach to the clustering, the scaled data was analyzed using the prcomp() function of RStudio using “factoextra” package developed by^22^. The “get eigenvalue” function of R-software was used to analyze the eigen-values, explained percentage variance by each PCA, and the cumulative variance percentage of each dimension. Also, the function “fviz_pca_biplot()” was used to plot the x-y axis of the PCA biplot.

## Results

### Analysis of variances results

The result of ANOVA revealed highly significant (p-value < 0.0001) differences among the studied genotypes for all the studied traits viz., ash, starch, tannin, nitrogen, protein and Beta-carotene (Table 2).

**Table 2:** Summary of ANOVA, maximum, minimum and mean results of 100 cassava genotypes.

Traits	MSG DF (99)	MSE (DF = 100)	CV (%)	Max	Min	Mean ± SD
Ash	5.22***	1.372	6.16	11.07	0.52	3.24 ± 0.83
Starch	4.95***	0.065	0.35	77.79	70.42	73.47 ± 0.18
Tannin	0.097***	0.00145	11.10	1.34	0.03	0.34 ± 0.027
BC	18.84***	1.762	12.50	18.91	6.23	7.68 ± 0.14
Protein	0.048***	0.0108	6.64	1.94	1.17	1.56 ± 0.073
Nitrogen	0.0012***	0.017	6.62	0.31	0.18	0.25 ± 0.012
MC	104.84***	2.97	5.49	82.62	28.37	53.82 ± 3.21
DM	155.40***	2.69	5.81	75.93	21.85	46.19 ± 2.25
OM	146.88***	9.54	5.75	71.67	18.55	42.96 ± 1.46

MSG=mean square of genotypes, MSE=mean square of error, CV=coefficient of variation, DM = dry matter, OM=organic matter, MC=moisture contenet, BC=beta carotene.

The mean separations of the selected studied genotypes are summarized in Table 3. The ash content ranged between 0.52% and 11.1%. The maximum ash content of 11.1% was recorded for genotype G103, followed by 10.85% for genotype G100. The genotypes G75 and G69 showed the minimum ash contents of 0.52 and 0.53%, respectively. Regarding the tannin contents of the studied genotypes with the highest (1.34 mg/100 g) tannin content was recorded on the genotype G86; while the lowest (0.02) was recorded on the genotype G82 (Table 3).

The maximum and minimum starch content of the studied genotypes was 77.99 and 70.22%, respectively, with a mean of 73.5%. The largest amount of starch content was obtained from genotype G33 (78.0%), followed by G29 (77.2%). The lowest starch contents were obtained on the genotypes G62 (70.42%) and G77 (70.44%) (Table 3).

The nitrogen and protein contents, which are the derivative of one another, ranged from 0.2 to 0.3% and 1.2–1.9%, respectively.

The beta carotene content ranged from 6.2 to 18.9 mg/100 g with a mean of 7.7 mg/100 g. The genotype G48 showed the highest beta carotene content, while the genotype G122 showed the lowest contents (Table 3).

**Table 3:** Summary of mean separation of studied biochemical content of cassava genotypes for the first three high yielder and the last two low yielder genotypes.

Geno BC (mg/g) Groups
G79 18.91 a
G106 17.82 ab
G35 16.75 abc
G116 6.42 v
G143 6.23 v
**Geno Ash (%) Groups**
G103 11.07 a
G100 10.85 a
G104 7.77 b
G69 0.53 r
G75 0.52 r
**Geno Starch (%) Groups**
G33 77.79 a
G29 77.22 b
G140 76.78 bc
G77 70.48 p
G62 70.42 q
**Geno Tannin (%) Groups**
G86 1.34 w
G3 0.99 v
G149 0.96 v
G80 0.04 a
G82 0.03 a
**Geno OM (%) Groups**
G128 71.67 a
G179 57.58 b
G149 56.53 bc
G64 24.95 j
G40 18.55 k
**Geno Protein (%) Groups**
G48 1.94 a
G99 1.89 ab
G106 1.88 ab
G142 1.20 st
G122 1.16 t
**Geno N (%) Groups**
G48 0.31 a
G106 0.31 ab
G99 0.31 ab
G142 0.19 lm
G122 0.19 m
**Geno MC Groups**
G86 82.62 a
G40 76.88 ab
G64 72.5 bc
G179 38.85 k
G128 28.37 l
**Geno DM (%) Groups**
G100 71.93 a
G128 71.63 b
G179 61.15 c
G40 23.12 kl
G86 17.38 l

### Multivariate data analysis

The data of targeted traits (ash, starch, tannin, moisture content, dry matter, organic matter, beta carotene, nitrogen and protein contents) were subjected to multivariate analyses such as correlation, principal component and cluster analyses, as follows.

#### Trait association analysis

**Fig. 1:**
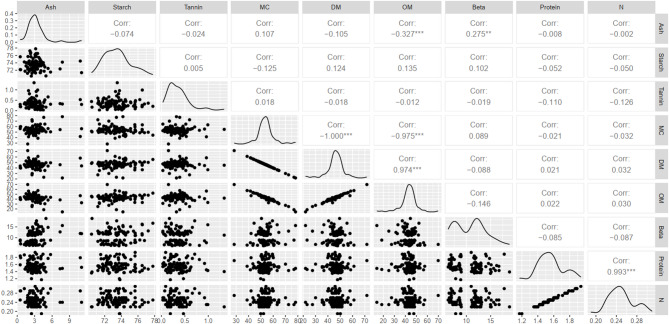
The correlation results of analyzed traits and data distribution of targeted traits.

From the result (Fig. 1), it can be observed that the data of the targeted traits followed normal distribution, except the tannin data, which was slightly skewed to the left, and the beta carotene data, which was slightly skewed to right from its mean.

The results of correlation analyses revealed highly significant and positive associations between organic matter and dry matter contents (0.98***) (Fig. 10). Ash content positively correlated with beta-carotene (0.275**) but strongly and negatively correlated with OM (-0.327***) However, starch positively correlated with OM (0.135) and DMC (0.124) and poor but negative correlated with protein.

#### Principal component analysis

The principal component analysis also confirmed the presence of significant variations among the studied genotypes in studied traits. The Principal Component Analysis (PCA) revealed that the first three dimensions with Eigen value greater than one explained 68% of total variation, whereas the first and second principal components (PCs) alone contributed to 56% of the total variation with the first and the second PCs each contributing to 33.3 and 22.7%, respectively, with cumulative contributions of 56% (Table 4). The traits that showed high loading values in the first dimension (PC1) were dry matter (0.99), moisture content (-0.99) and organic matter (0.99) contents (Table 5; Fig. 3). In the second dimension, nitrogen and protein content contributed high loading values of 0.99 and 0.98, respectively (Table 4). Ash and starch contents showed high negative (-0.8) and high positive (0.64) associations, respectively, with PC3 (Table 4).

**Table 4:** Contribution summary of the 1st three dimension with eigen value greater than one.

Traits	Dim1	Dim2	Dim3
Ash	-0.04	-0.01	**-0.80**
Starch	0.07	-0.09	**0.64**
Tannin	0.06	-0.22	0.12
Moisture content	**-0.99**	-0.03	0.08
Dry matter	**0.99**	0.03	-0.08
Organic matter	**0.99**	0.03	0.07
Beta-catotene	0.14	-0.20	-0.04
Protein	-0.06	**0.98**	0.04
Nitrogen	-0.05	**0.99**	0.04
Eigen value	3.00	2.00	1.00
% variance	33.7	22.7	11.6
% cumulative variance	33.3	56.40	68.00

### Genotypes by traits association analysis

**Fig. 2:**
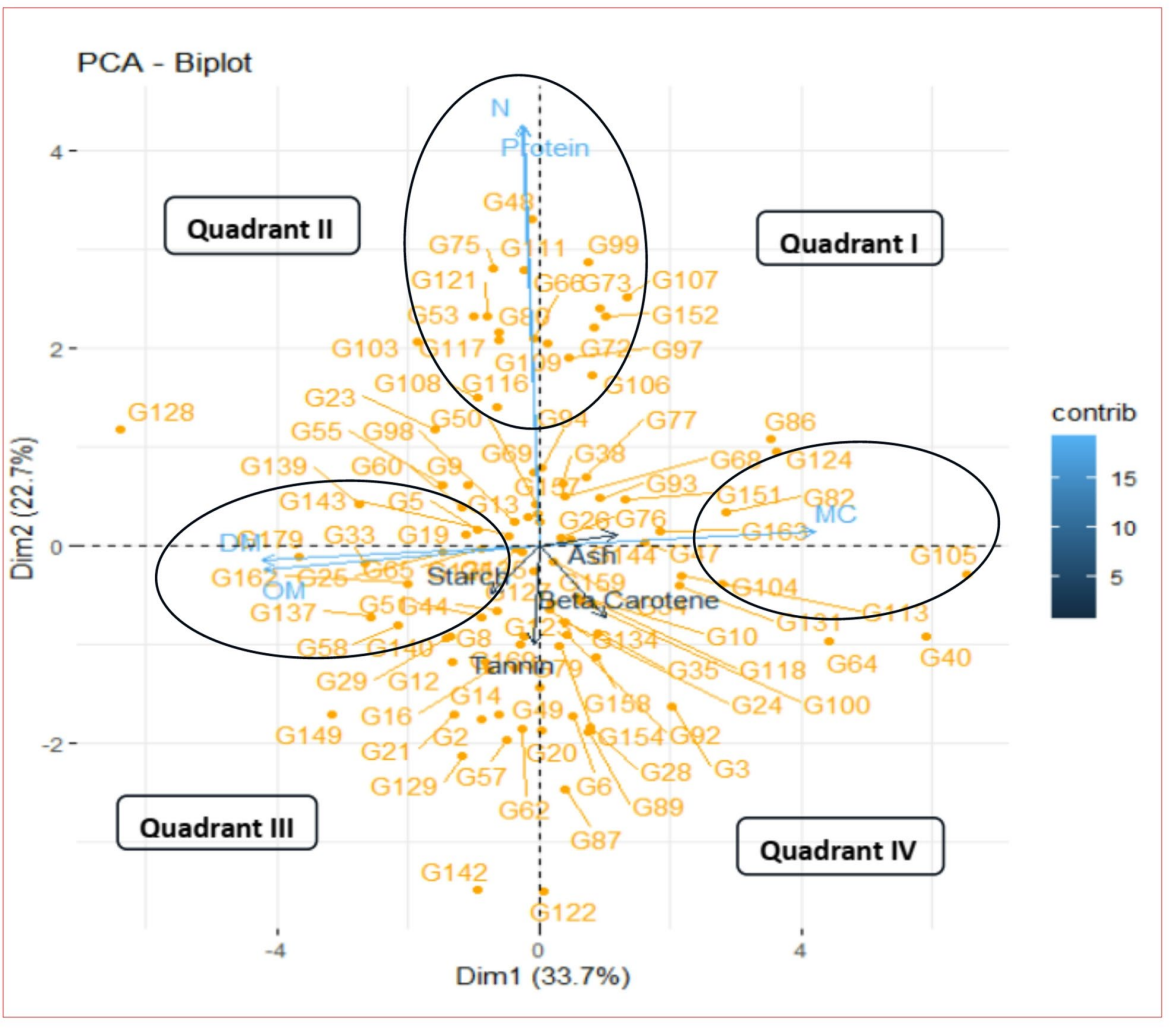
The biplot of PCA detecting contribution of the concerned traits and the study genotypes.

From the biplot (Fig. 2) result, the vectors of traits that fall in the same sector are positively correlated, whereas the vectors that fall in different sectors are negatively correlated. In a similar manner, the traits and genotypes found in the same direction have positive interrelationship and vice versa. Accordingly, genotypes found in quadrant I, especially, G179, G33, G19, G25, 162, G65, G143, G44, G137, G58 and G143 showed strong correlation with dry matter, organic matter and starch contents, whereas genotype G163, G76, G26, G47, G144, G105, G82, G151, G124 and G68 showed strong association with moisture content, which contributed more loading values to the first PC and to the total variations at the same time. The genotypes G48, G111, G66, G75, G80, G109, G94, G121, G116, G53, G50 G116, and G117 showed strong associations with nitrogen and protein contents (Fig. 2). So, Dim-1 is governed by dry matter, moisture content and organic matter while Dim-2 governed by nitrogen and protein contents.

### Cluster analysis

**Fig. 3:**
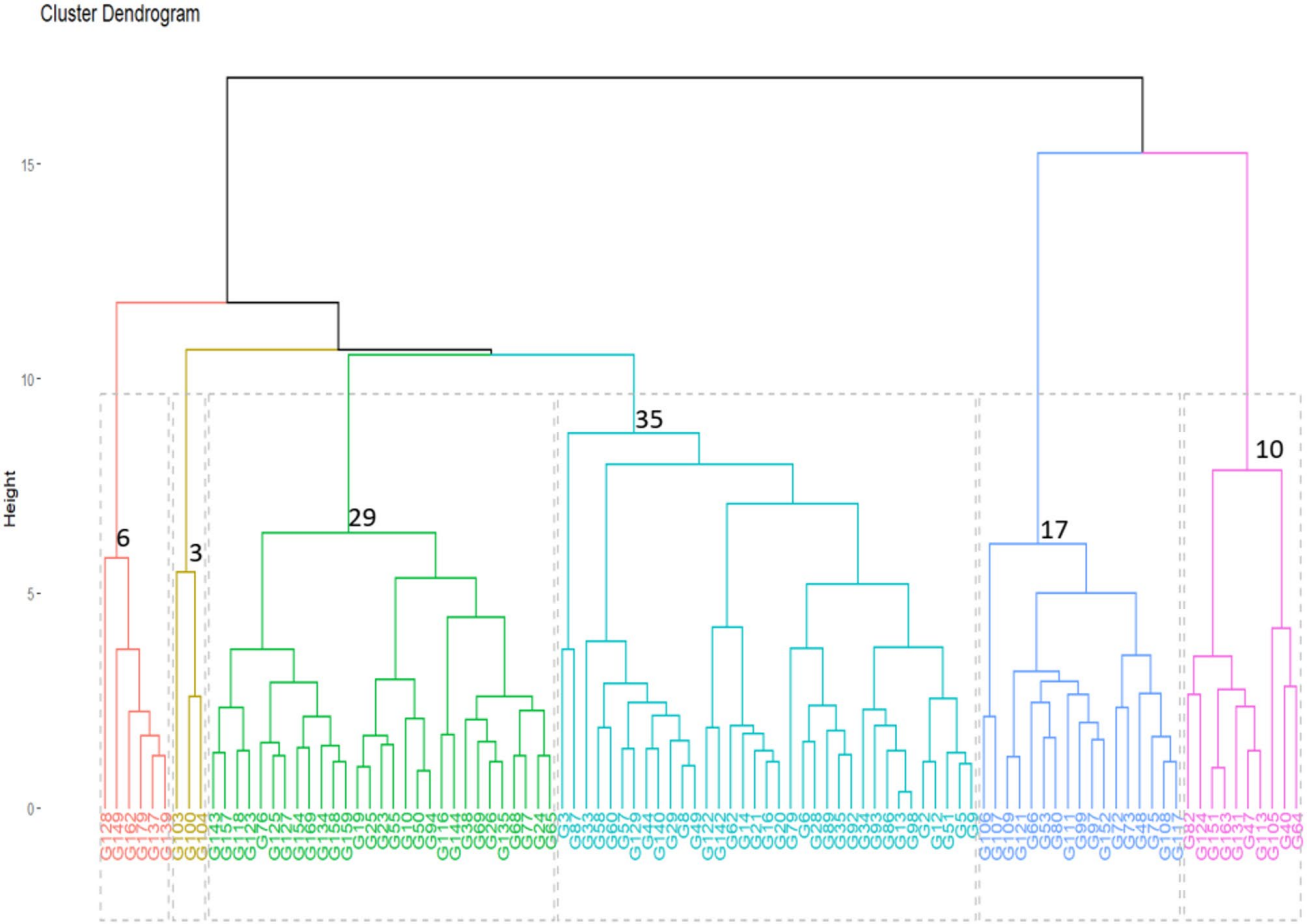
Dendrogram of clustered 100 selected cassava genotypes based on compositional analysis.

Cluster analysis grouped the studied materials into six distinct clusters (Fig. 3). Accordingly, the largest number (35) of the studied genotypes were grouped into cluster IV, comprising 35% of the studied genotypes, followed by cluster III, which contained 29 genotypes (29% of the studied genotypes). The least number of genotypes was found in cluster II, with only three genotypes (Table 4). Cluster mean analysis revealed that cluster I was characterized by the highest content of tannin, dry matter and organic matter, with the lowest ash content. The genotypes in cluster II were characterized by high ash and beta-carotene contents, while cluster III was characterized by high tannin content. The genotypes in cluster IV were characterized by high starch and beta-carotene contents, and low protein content. Genotypes in cluster V showed the highest crude protein and nitrogen contents, and the lowest tannin content (Table 6). On the other hand, genotypes grouped in cluster VI showed the highest moisture content and the lowest dry matter and organic matter contents.

**Table 5:** Lists and numbers of individuals genotypes grouped to each cluster.

Cluster	No. of genotypes	Lists of genotypes
Cluster I	6	G128, G137, G139, G149, G162, G179
Cluster II	3	G100, G103, G104
Cluster III	29	G10, G19, G23, G24, G25, G26, G38, G50, G55, G65, G68, G69, G76, G77, G94, G116, G118, G123, G125, G127, G134, G135, G143, G144, G154, G157, G158, G159, G169
Cluster IV	35	G2, G3, G5, G6, G8, G9, G12, G13, G14, G16, G20, G21, G28, G29, G33, G34, G35, G44, G49, G51, G57, G58, G60, G62, G79, G86, G87, G89, G92, G93, G98, G122, G129, G140, G142
Cluster V	17	G48, G53, G66, G72, G73, G75, G80, G97, G99, G106, G107, G108, G109, G111, G117, G121, G152
Cluster VI	10	G40, G47, G64, G82, G105, G113, G124, G131, G151, G163

**Table 6:** Mean of genotypes within cluster for studied traits.

Cluster	Ash	Starch	Tannin	MC	DM	OM	Beta-Carotene	Protein	*N*
Cluster I	2.48	73.55	**0.44**	40.57	**59.43**	**56.95**	6.88	1.56	0.25
Cluster II	**9.77**	72.73	0.39	50.95	49.22	39.45	**12.17**	1.60	0.26
Cluster III	2.59	72.73	0.37	53.84	46.16	43.57	9.68	1.56	0.25
Cluster IV	3.23	**74.40**	0.33	52.12	47.88	44.65	**12.65**	1.45	0.23
Cluster V	3.04	73.30	0.30	54.56	45.44	42.40	10.42	**1.83**	**0.29**
Cluster VI	3.86	72.89	0.32	67.25	32.75	28.89	9.38	1.53	0.24

**Fig. 4:**
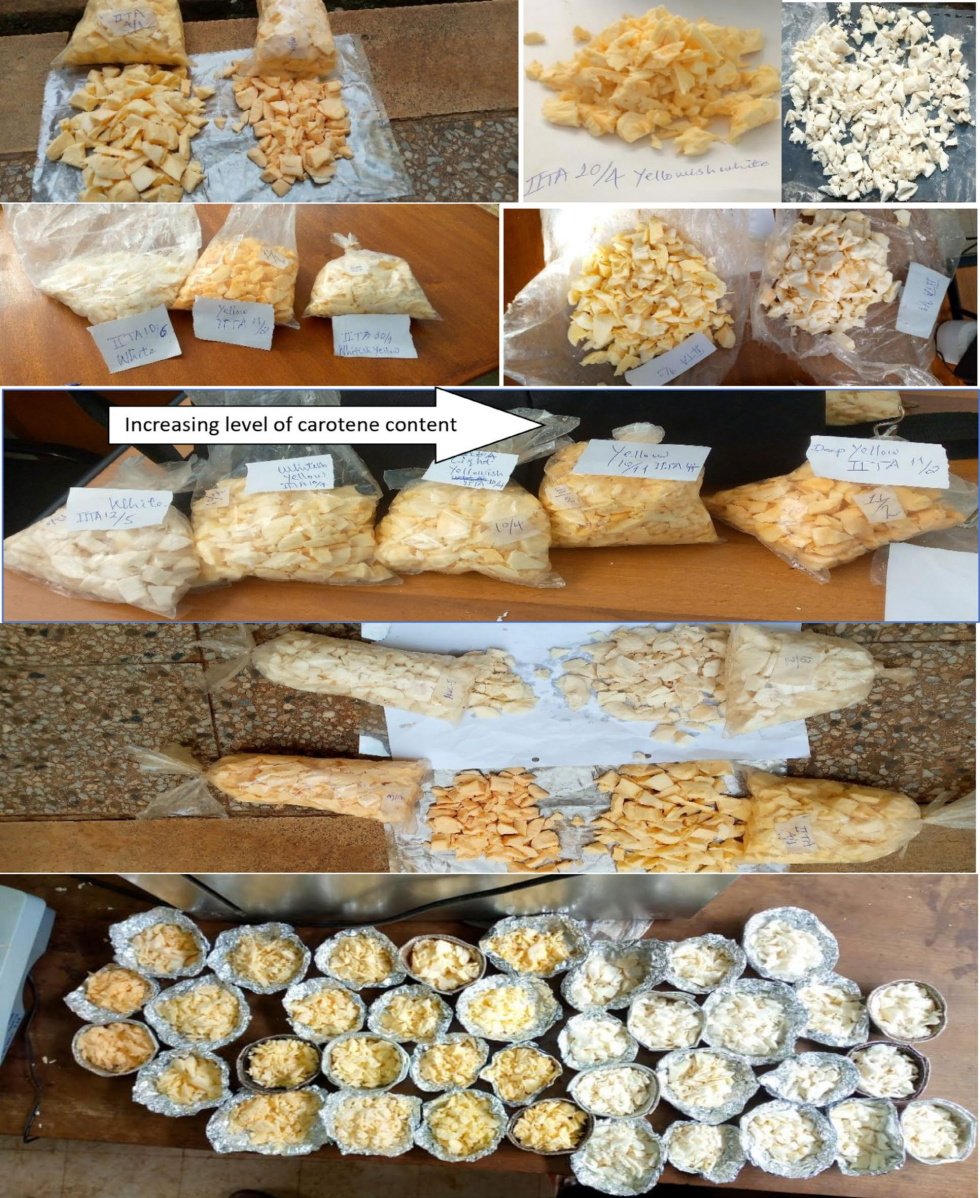
Different colors of storage root pulp of studied cassava genotypes sampled for oven drying at Jimma Agricultural Research Center.

## Discussion

### Analysis of variances of proximate composition

The observed highly significant differences among cassava genotypes for various nutritional composition traits namely ash, starch, tannin, dry matter (DM), organic matter (OM), moisture content (MC), beta-carotene, protein, and nitrogen content demonstrate the presence of considerable genetic variability within the studied germplasm. This broad variation suggests the potential for selective breeding targeting nutritional quality improvement. In alignment with this^16^, also reported highly significant differences among cassava accessions in Ethiopia for key traits such as DM, OM, ash, crude protein, tannin, and other biochemical properties, further affirming the diversity present in Ethiopian cassava populations.

The findings of the present study concerning protein, dry matter, and moisture content are consistent with the ranges reported by^5^, who found moisture content ranging from 45.9% to 85.3% and dry matter content from 29.8% to 39.3%, and^24^, who recorded a mean protein content of 2.35%. Additionally, the protein (1.03–2%), nitrogen (0.41–0.97%), and MC (56.67–70.19%) values observed in the current study are in agreement with those reported by^8^, supporting the reliability and reproducibility of these nutritional metrics across diverse studies.

On the other hand, ash content in the current study exceeded the values previously reported by^8^(0.4–1.7%) and Addisu et al. (2018)^24^ (1–2.56%). Ash, which indicates the total mineral content in food samples is an important quality marker in the food and flour industries^11^. According to^25^, the maximum allowable ash content in edible cassava root is 3%. The values reported in the present study, therefore, surpass this recommended threshold, which may reflect enhanced mineral accumulation but also warrants careful consideration for consumption standards where high ash levels may influence processing and consumer quality traits. Also, the result also indicated substantial genetic variability for mineral-related traits among the studied genotypes.

Similarly, other studies have shown significant genotype variation in cassava for nutritional traits. For instance^26^, identified wide ranges in starch, protein, and carotenoid contents among Latin American cassava accessions. Likewise^27^, reported high variability in beta-carotene and dry matter content among Ugandan cassava genotypes, reinforcing the global pattern of nutritional diversity in cassava germplasm collections.

The range of maximum and minimum percentage of ash (0.52–11.07), starch (70.2-77-79%), dry matter (21.85–71.63%) and moisture content (28.37–78.15%) found in this study widely varied from the finding of^3^ who reported ash (4.02–8.44%), starch (28.9–31.1), dry matter (93.56–95.27%) and moisture content (4.73–6.44%) were reported.

Also, protein content of the present study ranging from 1.11 to 2.00% is relatively in consistence with value cited by^16^, who reported 1.28 to 2.46% protein content despite it was far below protein content value of 17.56–31.15% reported by^3^. However, the lower protein values in the range 0.3–0.6% reported by^5^;^28^ were much lower than the result of our finding which might be due to management effect as well as varietal differences. However, the dry matter content of our study relatively equal to the finding of^6^ who reported mean of 31.20–43 based on oven drying and prediction equation methods. The higher dry matter and starch content of cassava indicates the opportunity of prolonged storability and easily ensure energy and food security^2^. It was reported that, genotypes have great influence on starch yield than environmental influences^11^. These wide compositional variations of our study with others’ finding might be due to either plant harvesting age, season of harvesting and/or management practice adhered to the plants. The protein content of our study genotypes falls within the range of protein content of cassava (1–2%) reported by^29^ which was higher than the range reported by^30^ but below the range (1.17–3.48%) reported by^31^ indicating the need to be supplemented with protein rich sources. The carotene content of sweet yellow cassava was reported to be in the range of 2.64 to 14.15 mg/g and the total beta-carotene content varied from 1.99 to 8.11 mg/g^32^ where the values of our study fall within these ranges.

Generally, the significant variation observed among the studied traits indicated that the presence of larger variations within the study genotypes indicating possibility of traits improvement through hybridization and selection.

### Multivariate analyses of biochemical content of cassava

Correlation analysis is a fundamental statistical tool in plant breeding and agricultural research that measures the strength and direction of the linear association between pairs of studied traits^33^. It plays a vital role in plant breeding as it gives insight to breeders in understanding the important traits^34^. Correlated characteristics have indicated association of the traits through genetic control which can either be genetic linkage or pleiotropic effect^35^. Organic matter has strong and positive correlation with dry matter content (0.98***) while strong and negative correlation with moisture content (-0.98***). This indicates that, as the organic matter content of the cassava root increases, the dry matter content also increases significantly but moisture content inversely decreases. Since dry matter represents the solid portion of the root (starch, fiber, proteins, etc.), higher OM reflects greater accumulation of these useful components. Therefore, genotypes with high OM are likely to have better nutritional and processing quality. On the other hand, the strong negative correlation between OM and moisture content means that roots with higher organic matter tend to have lower water content. This relationship suggests that selecting for higher organic matter will simultaneously improve dry matter content while reducing moisture content, which is desirable for traits like storage ability, processing efficiency, and overall root quality in cassava breeding program. The result was in agreement with the finding of^16^. Ash content strongly and positively correlated with beta-carotene (0.28**) but negatively correlated with OM (-0.33***). This implied OM includes carbohydrate, protein, lipids and other carbon-based compounds while ash contain is an inorganic minerals. So, the negative correlation suggested that compositional trade-off when increase in one composition reduces the other. On the other hand, positive association between ash and beta-caritene indicated the accumulation of mineral may enhanced the metabolic activity in carotenoid biosynthesis.

On the other hand, tannin and ash content of cassava root showed non-significant correlation with dry matter, moisture content and organic matter content in agreement with the report of^16^. The absence of significant correlations between tannin and ash content with dry matter, moisture content, and organic matter suggests that these traits are governed by distinct genetic and biochemical pathways. This independence provides an opportunity for simultaneous improvement of nutritional quality traits without compromising key agronomic and compositional characteristics in cassava.

Principal component analysis reduces the dimension of interrelated variables based on the correlated matrix explaining total standard variation^35^. The results of our study indicated dry matter and organic matter contents had positive and strong association with the first PCA while moisture contents had negative and strong correlation with the first PCA. In the second PCA, protein and N contributed more for observed variation. Accordingly, the first PCA was largely governed by dry matter, organic matter and moisture contents whereas the second PCA was governed by protein and N contents. The negative correlation of MC with DM and OM contents indicated the physiological trade-off in resource allocation^36^ which may be influenced by underlying genetic mechanisms including epistasis gene interaction.

Clustering analysis is used to categorize study materials into separate groups according to their similarity in traits of consideration^37^. Clustering is grouped cassava varieties with similar characteristics to display pattern and interrelationship between the samples and their nutritional properties^32^ where six cassava varieties grouped under three clusters, two varieties per each cluster. On the other hand, cluster analysis grouped 22 cassava tubers analyzed for sugar, protein, dry matter, anthocyanins and quinic acid into five categories^37^. The authors reported that, multivariate statistical methods particularly PCA are widely used to assess morphological and compositional traits of the crop. In consistence with our finding^16^, conducted study on 64 cassava genotypes for different biochemical composition and reported wider variations where studied materials grouped into six clusters. In general, multivariate data analyses indicated the presence of genetic relatedness and diversity available within the study panel as well as traits contributed to variation which guide breeders the way forward in parental selection for genetic improvement and conservation strategy.

## Conclusion and recommendation

The result of ANOVA revealed highly significant variation across the studied genotypes for all concerned traits. The result of ANOVA revealed highly significant variation across the studied genotypes for all considered traits. The magnitude of GCV of Tannin 128.8% with its corresponding heritability (H^2^) value of 98%, H^2−^value 97%, 84%, 83% and 82% of starch, DM, OM and MC with GAM vales of greater than 20% indicated majority of traits mainly influenced by high contribution of genetic effects providing opportunity of genetic gain through selection except for starch where mainly governed by environment. The result of multivariate analyses also witnessed that the studied genotypes were highly divergent where cluster analysis grouped genotypes into six main clusters and the first three PCA alone explained 68% of total variation where dry matter, moisture content and organic matter were contributors in dimension-1; and protein and nitrogen in dimension 2 respectively. Therefore, the study result can guide researchers to select parental materials with desirable traits of interest. Also, it is highly recommended if biochemical content including HCN content of the rest genotypes conducted; superior genotypes can be achieved. Popularization of the bio-fortified genotypes and effective utilization as parental material in breeding work can minimize the nutrient deficiency of our community.

## Data Availability

The data that support the findings of this study are available from corresponding author, but restrictions apply to the availability of these data, which were used under licence for the current study and so are not publicly available. The data are, however, available upon request and with the permission of the authors.

## References

[1] Rabbi, I. Y. et al. Genome-wide association analysis reveals new insights into the genetic architecture of defensive, agro-morphological and quality-related traits in cassava. *Plant Mol. Biol.*10.1007/s11103-020-01038-3 (2020). 32734418 10.1007/s11103-020-01038-3 PMC9162993

[2] Mégnanou, R. M. et al. Physico-chemical and biochemical characteristics of improved cassava varieties in Cote d’Ivoire. *J. Anim. Plant. Sci.* **5** (2), 507–514 (2009).

[3] Nadjiam, D., Ayessou, N. C. & Guissé, A. Physicochemical Characterization of Nine Cassava (Manihot esculenta Crantz) Cultivars from Chad. *Food Nutr. Sci.* **11**, 741–756. 10.4236/fns.2020.117053 (2020).

[4] Iglesias, C., Mayer, J., Chavez, L. & Calle, F. Genetic potential and stability of carotene content in cassava roots. Euphytica 94: 367–373, (2016) (1997).

[5] Montagnac, J. A., Davis, C. R. & Tanumihardjo, S. A. Nutritional value of cassava for use as a staple food and recent advances for improvement. *Compr. Rev. Food Sci. Food Saf.* **8**, 181–194 (2009). 33467798 10.1111/j.1541-4337.2009.00077.x

[6] Richardson, V. A. K. Quality characteristics, root yield and nutrient composition of six cassava (manihot esculenta crantz) varieties. *Gladstone Road Agricultural Centre Crops Research Report No*. 18. (2013).

[7] Sulistyo, J., Lee, J. S., Mammat, H. & Abdul, W. N. Nutritional value of fortified cassava flour prepared from modified cassava flour and fermented protein hydrolysates. 10.1063/1.4953504 (2026).

[8] Mohan, G. et al. Biochemical, mineral and proximate composition of Indian cassava varieties. *Int. J. Chem. Stud.* **7** (4), 1059–1065 (2019).

[9] Semman, N., Garedew, W. & Beyene, T. M. Proximate Composition Based Enset Diversity Study usingMultivariate Analysis in Sheka Zone, Southwest Ethiopia. *Res. J. Pharmacognosy Phytochem*. **11** (3), 167–174. (2019).

[10] Salvador, E. M., Steenkamp, V. & McCrindle, C. M. E. Production, consumption and nutritional value of cassava (*Manihot esculenta*, Cranz) in Mozambique: an overview. *J. Agricultural Biotechnol. Sustainable Dev.* **6** (3), 29–38. 10.5897/JABSD2014.0224 (2014).

[11] Temesgen, Z. T., Bakalo, B., Tamirat, H. & Medicinal Nutritional and Anti-Nutritional Properties of Cassava (*Manihot esculenta*): A Review. 10.5829/idosi.ajn.2019.34.46 (2029).

[12] Gul, K. et al. Chemistry, encapsulation, and health benefits of β-carotene - A review. *Cogent Food Agric.* **1**, 1018696. 10.1080/23311932.2015.1018696 (2015).

[13] Chandrasekara, A. & Kuma, T. J. Roots and Tuber Crops as Functional Foods: A Review on Phytochemical Constituents and Their Potential Health Benefits. *Int. J. Food Sci.* **3**, 3631647. 10.1155/2016/3631647 (2016). 10.1155/2016/3631647 PMC4834168 27127779

[14] Borku, A. W. Cassava (Manihot esculenta Crantz): its nutritional composition insights for future research and development in Ethiopia: Review. *Discover Sustain.*10.1007/s43621-025-00996-2 (2025).

[15] Abadura, N. S., Abush, T. A., Beye, T. M., Rabbi, I. Y. & Abtew, W. G. Characterization of cassava (Manihot esculenta [Cranz]) genotypes sourced from different regions in southwestern Ethiopia using morphological traits. *Genet. Resour. Crop Evol.*10.1007/s10722-025-02346-7 (2025).

[16] Berhanu, B. D., Derbew, B. Y., Tewodros, M. B. & Wosene, G. A. Biochemical Analysis of Cassava (*Manihot esculenta* Crantz) Accessions in Southwest of Ethiopia. *Hindawi J. Food Quality Article ID*. **9904103**, 13 pages. 10.1155/2022/9904103 (2022).

[17] Bradford, M. M. A rapid and sensitive method for the quantitation of microgram quantities of protein utilizing the principle of protein-dye binding. *Anal. Biochem.* **72**, 248–254. 10.1016/0003-2697(76)90527-3 (1976). 942051 10.1016/0003-2697(76)90527-3

[18] JIJE Determination of Beta Carotene in Foods. JIJE analytical testing service laboratory. Version number: 0. Page 1 of 4. Web site: www.jijelaboratory.com2016.

[19] Lopez-Hernandez, J., Gonzalez-Castro, M. J., Vazquez-Blanco, M. E., Oderiz, M. & Simal-Lozano, J. HPLC Determination of Sugars and Starch in Green Beans. *J. Food Sci. ResearchGate*. **59** (5), 1048–1049. 10.1111/j.1365-2621.1994.tb08186.x (2009).

[20] Makkar, H. P. S. Quantification of Tannins in Tree and Shrub Foliage: A Laboratory Manual. 10.1007/978-94-017-0273-7 (2003).

[21] De Mendiburu, F., Yaseen, M. & agricolae Statistical Procedures for Agricultural Research. R package version 1.4-0 (2020).

[22] Kassambara, A. & Mundt, F. Extract and Visualize the Results of Multivariate Data Analyses. Package ‘factoextra’. Version 1.0.7 (2020).

[23] Popat, R., Patel, R. & Parmar, D. Genetic Variability Analysis for Plant Breeding Research. Package version 0.1.0. 2020-09-29 08:30:02 UTC (2022).

[24] Addisu, S., Teshome, A. & Kassaye, T. Nutritional composition and sensory acceptability of cassava based composite flours. *Afr. J. Food Agric. Nutr. Dev.* **18** (1), 13131–13148. 10.18697/ajfand.81.16920 (2018).

[25] Wambua, M., Mulwa, R., Nduko, J. M., Matofari, J. & Nutritional Composition Antinutritive Compounds, and Starch Properties of Flour from Cassava Varieties Grown in Nakuru County, Kenya. 10.21203/rs.3.rs-3606733/v1 (2023).

[26] Chavez, A. L. et al. Variation of quality traits in cassava roots evaluated in landraces and improved clones. *Euphytica* **143** (1–2), 125–133. 10.1007/s10681-005-3057-2 (2005).

[27] Tumwegamire, S., Rubaihayo, P. R., Melis, R., Shanahan, P. & Benesi, I. R. M. Genetic variation in cassava for β-carotene and dry matter content in selected African germplasm. *Euphytica* **180**, 165–173. 10.1007/s10681-011-0354-7 (2011).

[28] Emmanuel, O. et al. Chemical composition and cyanogenic. *Int. Food Res. J.* **19** (1), 175–181 (2012).

[29] Adugna, B. Review on Nutritional Value of Cassava for Use as a Staple Food. *Sci. J. Anal. Chem.* **7** (4), 83–91. 10.11648/j.sjac.20190704.12 (2019).

[30] Menegucci, N. C., Leonel, M., Fernandes, A. M., Silva, J. G. & Nunes, J. G. d.S. Biomass yield and chemical composition of the cassava plant over two vegetative growth cycles. *Acta Scientiarum Agron.* **46**, e63719 (2024).

[31] Afoakwa, E. O., Asiedu, C., Budu, A. S., Chiwona-Karltun, L. & Nyirendah, D. B. Chemical composition and cyanogenic potential of traditional and high yielding CMD resistant cassava (*Manihot esculenta* Crantz) varieties. *Int. Food Res. J.* **19** (1), 175–181 (2012).

[32] Carvalho, L. M. J. et al. Retention of total carotenoid and b-carotene in yellow sweet cassava (Manihot esculenta Crantz) after domestic cooking. *Food Nutr. Res.* **56**, 15788. 10.3402/fnr.v56i0.15788 (2012). 10.3402/fnr.v56i0.15788 PMC3314373 22468142

[33] Gemeda, A. D. & Gurmu, G. N. Correlation Analysis on Yield and Yield Related Components in the Chickpea Genotypes under Irrigation of North East Ethiopia. *J. Plant. Biol. Crop Res.* **7** (2), 1101 (2024).

[34] Sivan, S. et al. Correlation studies and path analysis on root traits contributing to nitrogen use efficiency in cassava genotypes. *Pharma Innov. J.* **11** (11), 1040–1042 (2022).

[35] Maia, M. C. C. et al. Principal Component and Biplot Analysis in the Agro-industrial Characteristics of *Anacardium* spp. 10.19044/esj.2019.v15n30p21 (2019).

[36] Climent, J. et al. Trade-offs and Trait Integration in Tree Phenotypes: Consequences for the Sustainable Use of Genetic Resources. *Curr. Forestry Rep.* **10**, 196–222. 10.1007/s40725-024-00217-5 (2024).

[37] Chen, X., Zhang, L., Lin, X., Skalomenos, K. A. & Chen, Z. A clustering-based analysis method for simulating seismic damage of buildings in large cities. *ELSIEVER Eng. Struct.* **307**, 117860 (2024).

